# On the Use of Tobacco in Hernia, &c.

**Published:** 1828-08

**Authors:** James Wallace

**Affiliations:** Assistant Surgeon, R.N.


### HERNIA.

### *On the Use of Tobacco in Hernia, &c.* By James Wallace, Assistant Surgeon, R.N.

Along with the taxis, there are three great remedies which we have recourse to in strangulated hernia, before we have recourse to the operation. These are blood-letting, the warm bath, and tobacco; and all these tend towards the same end, namely, the production of syncope, or a state nearly approaching to it, by which, when we have the muscular power completely relaxed, we are enabled often to reduce the hernia without any difficulty. Each of the remedies has been found efficient; and, in the general, it is perhaps immaterial which of them we choose to gain our end by, provided that we do gain our end. However, it is necessary sometimes to make a choice, and occasionally, even amid them all, we are rather at a loss what to do. For example, in a debilitated patient, we should be loathe to take away blood, and would rather in this case have recourse to the bath. The bath, however, is not always to be had, or it is not to be had conveniently, or, when it is had, it may fail in its effect, and then of course we are thrown upon the tobacco. But the tobacco, although acknowledged to be a powerful remedy, is, in the way in which it is generally administered, rather an unmanageable one; and, as its use has occasionally been followed by alarming and even fatal consequences, it is dreaded by many, and has of late fallen much into disrepute. We are in the habit of throwing it into the rectum, either in a state of vapour or solution; and, if we throw in too much (and, unfortunately, we can never properly regulate the quantity,) dreadful sickness, and even death itself, may ensue; and, if we throw in too little, no effect will be produced. Therefore, it has been condemned by many as a remedy hardly admitting of use, and they tell us to try all other methods before this; for it would seem that the good which it may occasionally do is more than counterbalanced by the evil.

Now, it has occurred to me that we might use the tobacco in a much simpler and easier way, by which we might get the full benefit of its efficacy, while we should run no risk of working mischief with it: that, merely by changing the mode of its administration, instead of being a dernier resort, we might make it a first and general remedy. The plan I propose is—to give it by the mouth instead of the rectum; in plain words, to give the patient a pipe, and make him smoke, as if he were smoking for enjoyment, until he became thoroughly sick. Thus we could manage it exactly as we wished. We could make him perfectly sick, and relax his system completely, without being the least in danger of carrying the relaxing process too far, while we should save the time which is occupied in preparing the vapour or solution, and the trouble which is experienced in giving them. In ten or fifteen minutes, or less, the full effect would be produced, and then we might set to the taxis again with some hopes of success. I venture, therefore, to suggest, in cases of strangulated hernia, where bleeding is contra-indicated, and where the warm bath cannot readily be had, or where these two remedies, although tried, have failed to produce the effect required (which will sometimes happen), that we have recourse to the tobacco in this way. It is certain that bloodletting, in all cases where it is admissible, is the best measure which we can put in practice: first, because not only may it produce the effect which we are anxious to obtain for the reduction of the hernia, namely the syncope, but, in plethoric habits, it may greatly lessen the risk of after-inflammation. And, perhaps, next to bloodletting, we might in general make trial of the bath, if the bath can be got at once, and of the proper temperature. But if there is to be the least difficulty in getting it, so that we are to be in danger of losing time, or if we are likely to get it so inconveniently as not to give it a fair chance for its effect, then I think we had better let it alone: by giving the patient the tobacco, probably in less time than would have been occupied in preparing the bath, we might have him sickened, and his hernia returned. I am not an advocate for waiting long in the trial of any remedy for the reduction of hernia, for I conceive that the majority of patients are lost by unduly delaying the operation. If we do not succeed after a few trials,—after the trials of perhaps two or three hours, (symptoms being urgent,) I am quite sure that the sooner we perform the operation the better. But we always can, and we always should, if we see the patient soon after the occurrence of the complaint, while the parts are yet to be handled without giving pain, and there seems to be no immediate risk of dangerous inflammation, put in practice some of the means which have been mentioned; and, of course, that measure which is most convenient, and at the same time efficacious, will be chosen at once by the prudent practitioner.

It may be said that this remedy will not suit in many instances; that to those who are in the habit of using tobacco, it will be of no value. This is quite true. To the professed smoker, the remedy cannot be applied: but professed smokers will always be few in comparison with the mass of society, and therefore, in the greater number of cases, at any rate, this remedy will be applicable. Young people do not in general smoke; neither do females; only a few men smoke; so we may safely say that, in ten hernial cases, we shall not meet with more than one smoker. But, even allowing that we had to calculate in the contrary way,—allowing that out of ten cases we had nine persons that smoked, and only one that did not, still the tobacco would have its value. One life saved even out of a thousand is valuable; and a remedy which will cure or give relief even in an occasional case, should not be lost sight of.

Even on the score of delicacy there is a great point gained, if the plan which I now propose be found to answer. In desperate cases, I would not have any one stand about means, moreover, even an indelicate measure, when properly proposed and rightly gone about, becomes very different from what, at first thought, it was deemed. Nevertheless, in young females especially, there must always be a reluctance to the use of the tobacco in the common way; and therefore, if we can introduce it with equal, or even more, facility and success by the mouth, surely that method ought to be adopted in preference.

I have further to observe, that I think the remedy might be found useful in some other complaints. In dislocations, for instance, in robust people, where great extension is required to overcome the action of the muscles, and where, even with all our extension, we shall sometimes be foiled, would it not be advantageous to make trial of this plan? In dislocation of the hip-joint, which is so extremely difficult to reduce, would the remedy not be likely to give great help? Bloodletting, and the warm bath also, have often been used as auxiliaries in these cases; but would not this be, upon the whole, a better remedy? We do not, in cases of simple dislocation, require, in general, to bleed the patient from the fear of inflammatory action supervening, but merely for the purpose of rendering him faint at the time; and, if we can produce this faintness by simple smoking, it will surely be better than by bleeding. We are to bleed, certainly, where it is necessary, but we are to spare bleeding also wherever we can; and if the pipe will do at any time for the lancet, we ought to substitute it. And as the warm bath, in cases of dislocation as in cases of hernia, may sometimes not be easily got, and when got may not be of much use, it may be necessary to substitute the pipe for it also. Tartrate of antimony is frequently given to answer the same purpose, and has often a very good effect; but it may happen that we may be in a situation where we have neither a lancet nor a bath, nor tartrate of antimony, at hand, and it is well to know that we can have recourse to the simple plan of smoking, with some hope of doing good. If we can muster a pipe and a piece of tobacco, or a cigar, we may perhaps be able to do our work without loss of time, and without the help of other remedies.

Might the remedy not be of use in some other spasmodic and inflammatory diseases, especially when conjoined with other means. For example, in colic, and in obstinate constipation occurring from spasm, as in the colica pictonum,—in spasm of the biliary ducts, producing obstruction of bile,—in spasm of the ureter, or urethra, producing retention of urine and such diseases, might it not deserve a trial? At any rate it appears to me to be a thing worthy of remembrance, and perhaps in the hour of difficulty we may be glad to have recourse to it when other means fail.

*Portsmouth; May* 30*th*, 1828.

---

PIPERINE.

